# Donations to College Library

**Published:** 1879-11-20

**Authors:** 


### *DONATIONS TO COLLEGE LIBRARY—LIBERALITY OF LINDSAY & BLAKISTON, PHILADELPHIA.*

The following works have been generously contributed to Southern Medical College Library by Messrs. Lindsay & Blakiston, to-wit:

Manual of Midwifery, by Meadows; Diseases of Women, by Gallabin; Therapeutic Forces, by Dr. Mays; Practical Surgery, by Mears; Index of Diseases and their Treatment, by Tanner; Pennsylvania Hospital Report; Naval Hygiene, by Wilson; Lectures on Practical Surgery, by Toland; Diseases of Children, by Hillier; Reports on Medicine and Surgery, Sydenham Society; Anæsthetic Manual, by Turnbull; Diseases of the Stomach, by Habershon; Atlas of Human Anatomy, by Godlee; Summer and its Diseases, by Wilson; Epilepsy, Pains, Paralysis, by Radcliff; Lacerations of the Perineum, by Agnew; Chloroform, its Action and Administration, by Sansom; Complimentary Dinner to Prof. Gross.

